# Effect of Coriolus Versicolor Polysaccharide-B on the Biological Characteristics of Human Esophageal Carcinoma Cell Line Eca109

**DOI:** 10.7497/j.issn.2095-3941.2012.03.002

**Published:** 2012-09

**Authors:** Dao-feng Wang, Ning Lou, Xiao-dong Li

**Affiliations:** 1 State Key Laboratory of Oncology in South China,; 2 Department of ICU,; 3 Department of Thoracic Surgery, Cancer Center of Sun Yat-sen University, Guangzhou 510060, China

**Keywords:** esophageal carcinoma, CVPs-B, proliferation, apoptosis

## Abstract

**Objective:**

To investigate the effect of Coriolus versicolor polysaccharide-B (CVPs-B) on the biological characteristics of human esophageal carcinoma cell line Eca109 *in vitro*.

**Methods:**

The cells of experimental group (EG) were cultured in DMEM with 10% FCS and 150µg/mL CVPs-B, the cells of control group (CG) were cultured in DMEM with 10% FCS without CVPs-B. MTT reduction assay was performed to detect the effect of CVPs-B on the proliferation of Eca109 cells after the compound was administrated in varying concentrations. The living conditions of the Eca109 cells were determined using trypan blue exclusion. Then, cell growth curves were drawn. Flow cytometry was performed to detect the effect of CVPs-B on the apoptosis and cell cycle of Eca109.

**Results:**

In comparison with the CG, a marked decrease in the proliferation of Eca09 cells was observed in the EG, after incubation with CVPs-B. The survival rate of Eca09 cells decreased as the time of CVPs-B incubation prolonged. Comparing the cell cycles and apoptotic rates between the two groups, the proportions of cells in the G_0_/G_1_, S, and G_2_/M phases in the EG were found to be (68.4±3.7)%, (13.9±2.1)%, and (17.7±1.4)%, respectively, after 24 h incubation with CVPs-B. The cells had an apoptotic rate of (9.7±0.7)%. On the other hand, the proportions of the G_0_/G_1_, S, and G_2_/M cells of the CG were found to be (53.9±3.6)%, (26.6±2.8)%, and (19.5±2.3)%, respectively, with an apoptotic rate of (5.7±1.4)%. In comparison with the CG cells, significant cell growth in the G_0_/G_1_ phase was observed in the EG (*P*<0.05). Furthermore, a significant decrease in the number of cells in the S phase was observed (*P*<0.05) in the EG.

**Conclusions:**

CVPs-B can inhibit proliferation and enhance apoptosis of Eca109 cells and may be useful in the treatment of esophageal carcinoma.

## Introduction

Esophageal carcinoma (EsCa) is a common malignancy of the digestive tract that has rapid progression and easy metastasis. EsCa cells proliferate at early stages and infiltrate the surrounding submucosa and muscular layer. Thus, EsCa cells encroach the para-esophageal tissues, resulting in difficulties during radical excision. Previous findings^[^^1^^]^ have revealed that the biological behaviors of EsCa cells, such as proliferation and apoptosis, have mechanisms similar to their inflammatory reaction, which is affected by the inflammatory chemotactic factor CXCL12/CXCR4. As a biological immunomodulator, water-soluble Coriolus versicolor polysaccharide-B (CVPs-B) extracted from wild Turkey-tail not only regulates organic immunity and cellular oxidation, but also exhibits strong functions in regulating cell inflammation. Consequently, this mechanism regulates the pathway of the inflammatory chemotactic factor^[^^2^^]^. In this study, the effect of CVPs-B on the proliferation, apoptosis, and cycle of EsCa cells was observed by applying various concentrations of CVPs-B to the EsCa cell line Eca109.

## Materials and Methods

### Materials

This study utilized the following equipment and reagents: CO_2_ gas incubator (SHELD-M2300, USA), ELISA reader (micro-plate scanning spectrophotometer) (BIO-TEK, USA), flow cytometer (Becton Dickinson, USA), inverted microscope (Olympus, Japan), DMSO (Sigma, USA), MTT (Sigma, USA), 96-well culture plate (Corning, USA), 6-well culture plate (Corning, USA), FBS (Hangzhou Ever Green Organism Engineering Materials Co., China), and DMEM (Gibco, USA). The human esophageal squamous cell carcinoma (ESCC) cell line Eca109 was provided by the Laboratory of Center for Cancer Treatment and Prevention, Sun Yat-sen University.

### Preparation of CVPs-B

The CVPs-B was extracted from wild Turkey-tail and then purified in the State Key Laboratory, Cancer Center of Sun Yat-sen University, Guangzhou, by using high-performance liquid chromatography (HPLC). Proteins, polypeptides, and nucleic acids in CVPs-B were removed, leaving a polysaccharide with a relative molecular mass of 39 000.

### Cell culture

The Eca109 cells were cultured in an incubator at 37°C with 5% CO_2_ and saturated humidity. The culture medium contained DMEM with 10% FBS. The cells were used at 85% fusion in the cell culture. The culture medium was discarded at the logarithmic phase of the EsCa cells, and then digestion with 0.25% trypsin was conducted until cell detachment. DMEM with 10% FCS was added to the cells via blowing to cease digestion. Centrifugation was then performed for 5 min at 1 000 *r*/min. The supernatant was discarded, and a new culture medium was added to the cells. A cell count was conducted using a cell-counting slide after the blowing of the homogeneous single cell suspension. The culture medium was used to adjust the concentration of the cell suspension. The cell suspension was then seeded to a 96-well plate at 100 µL/well and was cultured at a saturated humidity of 5% CO_2_ and 37°C for 24 h to ensure the adherence of cells to the well walls.

### MTT assay

Cells were cultured in an incubator for 48 h and then taken out. The culture medium in the well was discarded and then 100 µL of new culture medium with varying concentrations of CVPs-B was added into the medium. The various concentrations of CVPs-B are as follows: 0, 50, 100, 150, and 200 µg/mL. In the experiment, three wells were set up for each concentration. The final DMSO concentration in each well was lower than 3‰. The cells were cultured in a 96-well incubator for another 48 h, and then 10 µL of 10 mg/mL MTT solution was added to each well. Cells were allowed to react in the incubator for 4 h and were then taken out. The solution in the wells was discarded. Then, 100 µL of DMSO was added to the cells, and agitation was performed. The wavelength of maximum absorbance was found to be 490 nm by using ELISA. The experiment was repeated twice, and the mean value of the three experiments was taken as the result. Cell growth curves were drawn with CVPs-B concentrations as the abscissa and absorbances as the ordinate.

### Trypan blue exclusion

The cells were cultured in an incubator for 48 h and were then taken out. The culture medium in the well was discarded, and then 100 µL of culture medium with 150 µg/mL CVPs-B was added to the experimental group (EG). The following four culture periods were adopted: 0, 24, 48, and 72 h. Trypsinization was conducted to make the cells adhere to the well walls, and a single cell suspension was prepared. A 0.4% Trypan blue solution was added to the suspension (ratio of 9:1) for 5 min of staining. Viable cells were counted on a cell-counting slide. The experiment was repeated twice. The mean value of three tests was presented as the final result. Cell survival rate was calculated as follows: total viable cells/(viable cells + total dead cells)×100%.

### Flow cytometry

The Eca109 cells from the EG and the control group (CG) were cultured in DMEM with 10% FCS at 37°C and a saturated humidity of 5% CO_2_. The cells were then seeded on a 6-well plate for 24 h. The culture medium in the wells of EG was discarded, and then 100 µL of the culture medium containing 150 µg/mL CVPs-B was added. Cells were cultured in the incubator for another 72 h. Cells from both groups were harvested, rinsed twice with PBS, fixed with 70% glacial ethanol, and then left overnight at 4°C. Centrifugation was conducted for 5 min at 1000 *r*/min, and the ethanol was discarded. Propidium iodide was added to the cells, which were then cultured at 4°C for 30 min in the dark. Flow cytometry was performed to detect the cell cycle and apoptosis. The mean value of three experiments was presented as the result.

### Statistical analysis

The obtained data were verified using mean±SD and then processed using SPSS10.0 software. Student *t*-test was used to compare the means of independent samples. *P* values < 0.05 indicated significant differences.

## Results

**Figure 1:**
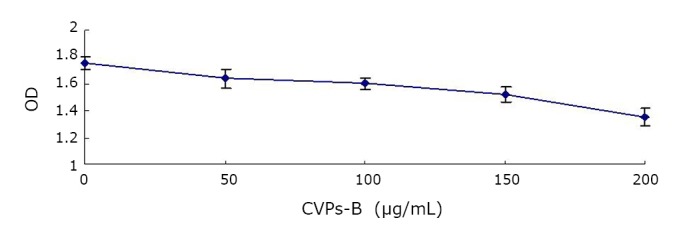
Effects of various concentrations of CVPs-B on the proliferation of esophageal cell line Eca109.

**Figure 2:**
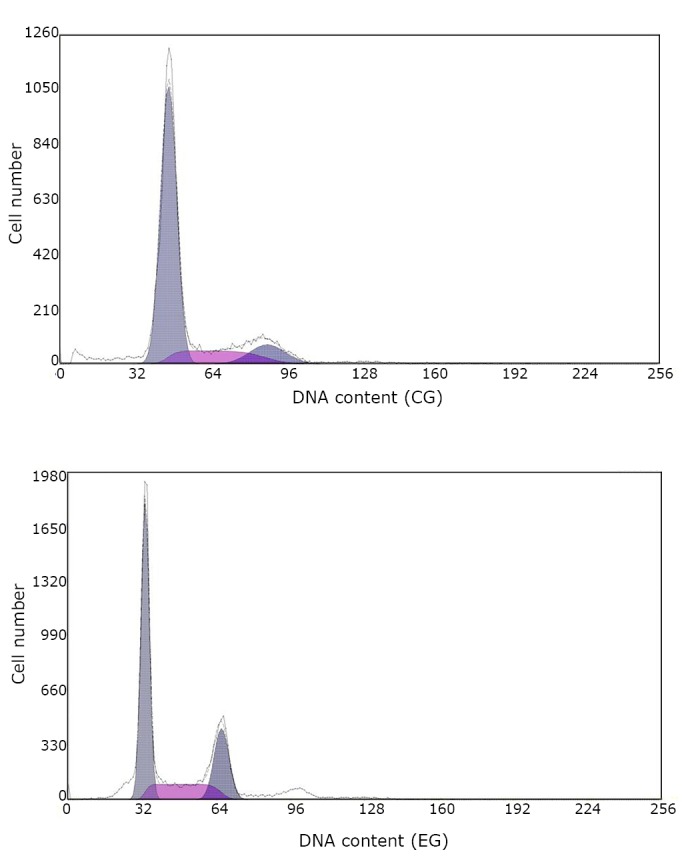
Changes in cell generation cycle and apoptosis of Eca109 cells in CG and EG.

The effects of various concentrations of CVPs-B on EsCa cell line Eca109 after 48 h of incubation are shown in **Figure 1**. The effects of CVPs-B on the survival rate of Eca109 are shown in **Table 1****.** The effects of CVPs-B on the cell cycle and apoptotic rate of Eca109 after 72 h of culture are shown in **Table 2** and **Figure 2**.

**Table 1:** Effects of CVPs-B on the survival rate of Eca109 cells.

Survival rate	CG (%)	EG (%)	*P*
o h	93.2±2.6	92.7±1.7	0.853
24 h	93.6±1.9	86.8±1.7	0.001
48 h	95.1±0.6	81.3±3.7	0.016
72 h	92.4±2.5	82.2±2.4	0.008

**Table 2:** Effect of CVPs-B on the cycle and apoptosis rate of Eca109 cells.

	EG (%)	CG (%)	*P*
G_0_/G_1_ phase	68.4±3.7	53.9±3.6	0.001
S phase	13.9±2.1	26.6±2.8	0.016
G_2_/M phase	17.7±1.4	19.5±2.3	0.263
Apoptotic rate	9.7±0.7	5.7±1.4	0.020

## Discussion

ESCC has the characteristics of high infestation and metastasis. Sub-mucosal invasion can easily occur at the early phase of tumor cells. Infiltration, outward encroachment, and distant metastasis may emerge via the mucous membrane of the esophagus, pervasive sub-mucous lymphatic, and vascular nets. Related studies^[^^3^^,^^4^^]^ revealed that the biological behaviors of EsCa cells, such as their carcinogenesis, progression, and metastasis, are closely related to the pathway of the inflammatory chemotactic factor. Related studies and reports on other tumors are also available^[^^5^^,^^6^^]^. CVPs-B, a kind of heparitin, is a protein-binding polysaccharide that may be extracted and purified from wild Turkey-tail via HPLC. Polypeptide and nucleic acid elements may be removed from the compound to increase the water solubility of the compound. Previous studies^[^^7^^,^^8^^]^ indicated that CVPs-B inhibits the expression of the osteopontin (OPN) gene. Moreover, CVPs-B can also down-regulate glycosaminoglycan (GAG) expression on the surface of macrophages, affecting the expression of the inflammatory chemotactic factor. The effect of CVPs-B on several biological features, such as EsCa cell viability and proliferation, was evaluated based on the interaction of CVPs-B with the inflammatory chemotactic factor. However, this mechanism remains uncertain because studies on the polysaccharide are empirical in nature. In the current study, the effect of the immunobiological modulator on the viability of proliferation, proliferation cycle, and apoptotic rate of EsCa cells was observed by applying various concentrations of CVPs-B to EsCa cells.

In the experiment, various concentrations of CVPs-B were allowed to react with the EsCa cells for 48 h. The number of EsCa cells observed in the EG decreased with increasing CVPs-B concentration. Moreover, the EG exhibited lower cell counts than the CG after incubation at varying lengths of time at a constant concentration of 150 µg/mL CVPs-B. In comparison with CG, the obtained cell counts from EG increased during the G_0_/G_1_ phase and decreased during the S phase after incubation with CVPs-B. These results suggest that CVPs-B reacted with ECA109 cells and increased the number of cells during the resting phase. Furthermore, the polysaccharide may reduce the number of cells during the proliferation phase, that is, the CVPs-B may inhibit the proliferative activity of Eca09 cells and induce the cells to proceed to the resting phase of cell growth faster. The results above are similar to the results of Lee et al.^[^^9^^]^ Previous findings^[^^1^^]^ demonstrated that the inflammatory chemotactic factor plays an important role in the progression and metastasis of EsCa. CVPs-B suppresses the expression of the OPN gene, resulting in the down-regulation of GAG expression on the cell surface. Moreover, a key premise binds to the chemotactic factor (SDF-1/CXCL12) and the GAGs on the cell membrane when the members of the chemotactic factor family realize the induction of large numbers of inflammatory cells into the inflammatory site during the inflammatory reaction^[^^8^^,^^10^^]^. Therefore, the action of CVPs-B on the accessibility of the chemotactic factor may be considered as one of the mechanisms in which CVPs-B inhibits the proliferation of EsCa cells and modulates their cell cycle.

In this study, the cell cycle rate in EG increased after incubation with CVPs-B compared with the CG. The mechanism of CVPs-B regulation on the genetic expression of macrophage OPN^[^^11^^]^ indicates that the regulation of CVPs-B on OPN gene expression is determined by the communication between the oxidation signals and the inflammatory signals in the cells (cross-talk) in the stress response system pathway, and not by the p38MAPK inflammatory signal pathway. Therefore, the regulation of CVPs-B on p38MAPK can be considered as one of the mechanisms of the modulator. Other pathways and homologous oxidative stresses signal the system to act on p38MAPK and initiate its regulatory function of apoptosis accordingly. Another related study^[^^12^^]^ stated that the inflammatory chemotactic factor CXCL12 may also up-regulate the transcription of the apoptosis-related gene Bcl-2 via the activation of p44/42MAPK. In the current study, the action of CVPs-B on CXCL12 and its modulation of the cell cycle via the inflammatory signal p44/42MAPK were assumed to be some of the mechanisms of CVPs-B.

CVPs-B can inhibit the proliferation and apoptosis of EsCa cells, regulate the cell cycle of EsCa cells, and induce more tumor cells to adopt their resting state faster. Further research is necessary to elucidate the related mechanisms and signal transduction system of CVPs-B.

## References

[1] Wang DF Lou N Zeng CG Expression of CXCL12 / CXCR4 and its correlation to prognosis in esophageal squamous cell carcinoma. Chinese Journal of Cancer 2009; 28: 154-158 (in Chinese) 19550128

[2] Lou N Wang DF Fang Y Effects of CVPs-B on macrophagocyte osteopontin-proteoglycan combined with oxidized low density lipoprotein. Chinese Pharmacological Bulletin 2009; 25: 1181-1184 (in Chinese)

[3] Sasaki K Natsugoe S Ishigami S Expression of CXCL12 and its receptor CXCR4 correlates with lymph node metastasis in submucosal esophageal cancer. J Surg Oncol 2008; 97: 433-438 18176915 10.1002/jso.20976

[4] Kaifi JT Yekebas EF Schurr P Tumor-cell homing to lymph nodes and bone marrow and CXCR4 expression in esophageal cancer. J Natl Cancer Inst 2005; 97: 1840-1847 16368946 10.1093/jnci/dji431

[5] Wei M Liang LZ Zhang CQ Correlation of CXCR4/CXCL12 over- expression to lymph node metastasis and chronic inflammation in cervical adeno-carcinoma. Ai Zheng 2007; 26: 298-302 (in Chinese) 17355795

[6] Pils D Pinter A Reibenwein J In ovarian cancer the prognostic influence of HER2/neu is not dependent on the CXCR4/SDF-1 signaling pathway. Br J Cancer 2007; 96: 485-491 17245339 10.1038/sj.bjc.6603581 PMC2360022

[7] Lou N Ma G Wang DF Effect of Coriolus versicolor polysacchar ide B on membr ane glycosaminoglycans and cellular glutathione changes in RAW264.7 macrophages exposed to angiotensin II. J South Med Univ 2007; 27: 1824-1826 18158993

[8] McCornack MA Cassidy CK, LiWang PJ. The binding surface and affinity of monomeric and dimeric chemokine macrophage inflammatory protein 1 beta for various glycosaminoglycan disaccharides. J Biol Chem 2003; 278: 1946-56 12411442 10.1074/jbc.M207440200

[9] Lee Y Gotoh A Kwon HJ Enhancement of intracellular signaling associated with hematopoietic progenitor cell survival in response to SDF-1/CXCL12 in synergy with other cytokines. Blood 2002; 99: 4307-4317 12036856 10.1182/blood.v99.12.4307

[10] Proudfoot AE Handel TM Johnson Z Glycosaminoglycan binding and oligomerization are essential for the in vivo activity of certain chemokines. Proc Natl Acad Sci U S A 2003; 100: 1885-90 12571364 10.1073/pnas.0334864100 PMC149928

[11] Lou N Ma G Wang DF Effect of Coriolus versicolor polysaccharides B on osteopontin mRNA expression in macrophages induced by angiotensin. Chinese Pharmacological Bulletin 2008; 24:1284-8 (in Chinese)

[12] Bonni A Brunet A West AE Cell survival promoted by the ras-MAPK signaling pathway by transcription-dependent and independent mechanisms. Science, 1999; 286: 1358-1362 10558990 10.1126/science.286.5443.1358

